# Efficacy of eltrombopag in thrombocytopenia after hematopoietic stem cell transplantation

**DOI:** 10.3906/sag-2111-36

**Published:** 2022-02-23

**Authors:** Ayşe KARATAŞ, Hakan GÖKER, Haluk DEMİROĞLU, Ümit Yavuz MALKAN, Mustafa VELET, Olgu Erkin ÇINAR, Batuhan ERDOĞDU, Mine KARADENİZ, Nilgün SAYINALP, Salih AKSU, İbrahim Celalettin HAZNEDAROĞLU, Osman İlhami ÖZCEBE, Yahya BÜYÜKAŞIK

**Affiliations:** Division of Hematology, Department of Internal Medicine, Faculty of Medicine, Hacettepe University, Ankara, Turkey

**Keywords:** Hematopoietic stem cell transplantation, bone marrow transplant, eltrombopag, prolonged isolated thrombocytopenia, secondary failure of platelet recovery

## Abstract

**Background/aim:**

Thrombocytopenia is a common complication following hematopoietic stem cell transplantation (HSCT). Eltrombopag has been used in thrombocytopenia treatment after HSCT in recent years. Herein, we present our experience of 25 patients treated with eltrombopag for post-HSCT thrombocytopenia.

**Materials and methods:**

Fifteen autologous hematopoietic stem cell transplantation (AHSCT) and 10 allogenic hematopoietic stem cell transplantation (allo-HSCT) recipients treated with eltrombopag for treatment of prolonged isolated thrombocytopenia (PIT) or secondary failure of platelet recovery (SFPR) in the stem cell transplantation unit of Hacettepe University Hematology Department between 2017 and 2021 were included in the study. The primary endpoint of this study is eltrombopag response in patients diagnosed with PIT or SFPR. Platelet count above 50,000/mm^3^ for five consecutive days without platelet transfusion was considered as eltrombopag response. Overall survival (OS) analyses were calculated based on the time between HSCT and death from any cause. The patients who were alive at the last follow-up were censored at this time for calculation of OS analyses.

**Results:**

AHSCT (66.7% (10/15)) and allo-HSCT (50% (5/10)) recipients responded to eltrombopag for the treatment of post-HSCT thrombocytopenia. There was no excess toxicity related to the eltrombopag use. The median response duration of allo-HSCT recipients and AHSCT recipients were 41 (13–104) days and 50 (7–342) days, respectively. There was a statistically significant OS duration difference between the responders and nonresponders in allo-HSCT and AHSCT recipients with p values of 0.005 and 0.02, respectively.

**Conclusion:**

Eltrombopag is promising for the treatment of thrombocytopenia after AHSCT and allo-HSCT in terms of efficacy and safety.

## 1. Introduction

Prolonged thrombocytopenia is a common complication following hematopoietic stem cell transplantation (HSCT). It occurs in 5%–37% of patients after allogenic stem cell transplantation (allo-HSCT) [1].

In various studies, it was shown that platelet count below 100,000/mm^3^ after the 100^th^ day of HSCT increases the transplantation-related mortality (TRM) [2,3]. The most common risk factors for post-HSCT thrombocytopenia are disease remission status, amount of infused CD34^+^ cells, graft-versus-host disease (GVHD), stem cell source, cytomegalovirus (CMV) infection, and drugs [4,5].

There is not known guideline categorizing patients with isolated thrombocytopenia after HSCT. Isolated thrombocytopenia after HSCT is mostly divided into two different categories: prolonged isolated thrombocytopenia (PIT) and secondary failure of platelet recovery (SFPR). Prolonged isolated thrombocytopenia is generally defined as a platelet count below 20,000/mm^3^ or persistence of the need for platelet transfusion beyond 60 days after HSCT; despite the other blood series engrafted. In addition, in some cases, the absence of primary platelet engraftment until the 35^th^ day after transplantation is considered as delayed thrombocyte engraftment [6]. SFPR is defined as a platelet count of less than 20,000/mm^3^ for seven consecutive days after primary platelet engraftment or a need for transfusion after platelet count exceeds 50,000/mm^3^ for seven consecutive days without transfusion [5]. Thrombocytopenia may also occur as a component of poor graft function (PGF) after HSCT. Options for PGF treatment include CD34^+^ stem cell boosts, mesenchymal cell infusion, and granulocyte-colony stimulating factors [7].

Approved treatment options for isolated thrombocytopenia following HSCT are limited to steroids and intravenous immunoglobulin after the elimination of reversible causes [8]. Eltrombopag, an oral thrombopoietin receptor agonist approved for use in aplastic anemia and refractory immune thrombocytopenia, has been used in thrombocytopenia treatment after HSCT in recent years [9,10]. Herein, we present our experience of 25 patients treated with eltrombopag for post-HSCT thrombocytopenia.

## 2. Materials and methods

Patients who received eltrombopag for treatment of PIT or SFPR in the stem cell transplantation unit of Hacettepe University Hematology Department between 2017 and 2021 were included in the study. Data of these patients were examined from the hospital database retrospectively.

It was confirmed that an informed consent form was obtained from all patients included in the study as a routine application of Hacettepe University Medical Faculty Hospital when they were admitted to the hospital. The approval was obtained from the local ethics committee with decision number of 2021/15-48.

The eltrombopag treatment was started at a dose of 25 mg/day. It was increased by 25 mg/day weekly, with a maximum dose of 150 mg/day when adequate response was not achieved.

The primary endpoint of this study is eltrombopag response in patients diagnosed with PIT or SFPR. Platelet count above 50,000/mm^3^ for five consecutive days without platelet transfusion was considered as eltrombopag response. Overall survival (OS) analyses were calculated based on the time between HSCT and death from any cause. The patients who were alive at the last follow-up were censored at this time for calculation of OS analyses.

## 3. Statistical analysis

The SPSS software version 25 (SPSS Inc., Chicago, IL, USA) was used for statistical analyses. The variables were evaluated using analytical methods (the Kolmogorov–Smirnov/Shapiro–Wilk’s tests) to determine whether they are normally distributed or not. The distribution of continuous data was examined. Mean (±standard deviation) and median (minimum-maximum) values were given for normally distributed continuous and nonnormally distributed variables, respectively. Categorical data was analyzed using chi-square or Fisher’s exact test. Survival analyses were done using Kaplan–Meier test. The effect of eltrombopag response on OS was investigated using the log-rank test. The p values < 0.05 were considered statistically significant.

## 4. Results

Twenty-five patients were included in the study. Median age was 53.3 (23.9–65) years. Nineteen patients (76%) were treated with eltrombopag for PIT and six patients (24%) for SFPR. Fifteen of the patients (60%) underwent autologous hematopoietic stem cell transplantation (AHSCT), and ten of the patients (40%) underwent allo-HSCT. Baseline characteristics of the patients are outlined in Table 1 .

Median follow-up was 356 (20–1515) days. Fifteen out of twenty-five patients (60%) responded to eltrombopag treatment. Median response duration was 41 (7–342) days. The cumulative data of all patients receiving eltrombopag is given in Table 2 . The data of AHSCT and allo-HSCT recipients receiving eltrombopag are given in Table 3 separately.

Eltrombopag response rates of the allo-HSCT recipients according to gender, conditioning regimen type (myeloablative or reduced intensity), the indication of treatment (PIT or SFPR), presences of GVHD and CMV infection are given in Table 4 . Similarly, response rates of the AHSCT recipients according to gender, remission status, and an indication of treatment are given in Table 5. Response rates were not statistically different in terms of these parameters (p > 0.05).

At the end of the study, thirteen (52%) of the patients deceased: 60% (6/10) of allo-HSCT recipients and 46.6% (7/15) of AHSCT recipients.

**Figure 1:**
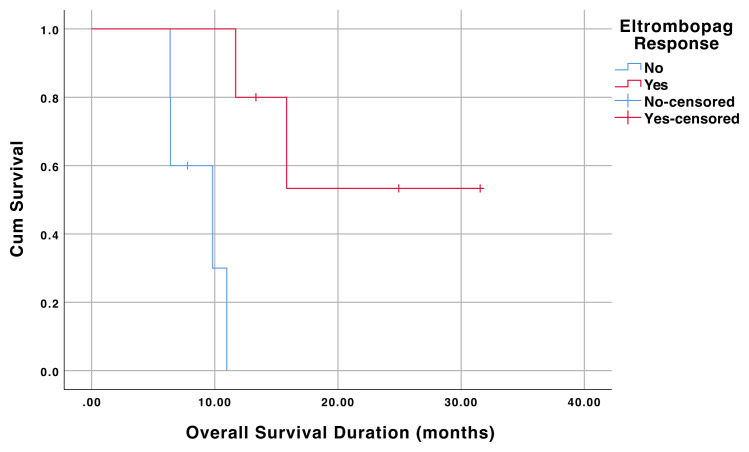
Overall survival of the allo-HSCT recipients that responded and did not respond to eltrombopag.

There was a statistically significant OS duration difference between the responders and nonresponders in allo-HSCT recipients (p = 0.005). The mean OS duration of the allo-HSCT recipients responded to eltrombopag was 23.3 (95% CI 14.9–31.8) months. The mean OS duration of the allo-HSCT recipients who did not respond to eltrombopag was 8.7 (95 CI % 6.7–10.8) months. OS curves of the allo-HSCT recipients that responded and did not respond to eltrombopag are shown in Figure 1 .

**Figure 2:**
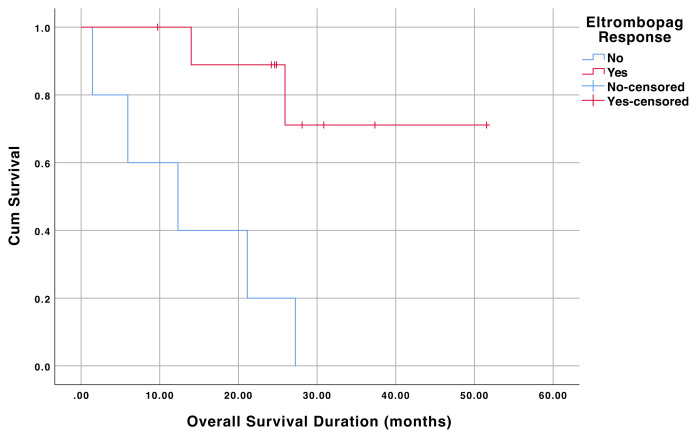
Overall survival of the AHSCT recipients that responded and did not respond to eltrombopag.

Similarly, in AHSCT recipients, OS was statistically significantly different between the patients that responded to eltrombopag or not (p = 0.02). The mean OS duration of the AHSCT recipients responded to eltrombopag was 42.8 (95% CI 32.4–53.2) months. The mean OS of AHSCT recipients who did not respond to eltrombopag was 13.6 (95% 4.3–22.9) months. OS curves of the AHSCT recipients that responded and did not respond to eltrombopag are shown in Figure 2.

## 5. Discussion

Thrombocytopenia and hemorrhage are common life-threatening complications following HSCT. In a retrospective study including 491 patients with a median follow-up duration of 33 months after HSCT, life-threatening bleeding was reported in 9.4% of the patients. Severe hemorrhages were found to be associated with especially thrombotic microangiopathy, GVHD, and prolonged thrombocytopenia [9,11]. Thrombotic microangiopathy, infections, drugs that may cause thrombocytopenia should be ruled out immediately after post-HSCT thrombocytopenia is diagnosed [12].

Treatment options for thrombocytopenia after HSCT are limited to steroids and intravenous immunoglobins [8]. Although eltrombopag is not approved for post-HSCT thrombocytopenia, it has been used for this indication in recent years. There are many publications reporting its safety and efficient use, especially after allo-HSCT [13–16].

In a large multicenter study from Spain, Bento et al. [13] reported the results of 86 patients treated with platelet receptor agonists for prolonged thrombocytopenia after HSCT. They reported 72% of the patients had responses in a median time of 66 days. In another publication, it was reported that the platelet count exceeded 50,000/mm^3^ in 52.3% of 38 patients treated with eltrombopag for thrombocytopenia after haploidentical allo-HSCT [14].

Yuan et al. [15] reported the results of 13 allo-HSCT recipients treated with eltrombopag. The overall response rate was 62% and the median response time was 33 days.

Tanaka et al. [16] investigated the efficacy of eltrombopag in a study that includes five patients diagnosed with PIT and seven patients diagnosed with SFPR. They reported the eltrombopag responses as 60% and 72% for PIT and SFPR patients, respectively.

There are few studies, mostly case reports or case series, about the efficacy of eltrombopag for the treatment of thrombocytopenia after AHSCT [17,18]. Raut et al. [19] observed eltrombopag efficacy on two allo-HSCT and ten AHSCT recipients, with a median treatment duration of 29 days. They reported an increase in platelet count in all patients, with a median of 36,000/mm^3^.

To the best of our knowledge, our study is one of the studies that reflects the experience of eltrombopag in the largest number of AHSCT recipients in the literature.

In the present study, 66.7% (10/15) of AHSCT and 50% (5/10) of allo-HSCT recipients responded to eltrombopag treatment. The median response duration of allo-HSCT recipients and AHSCT recipients were 41 (13–104) days and 50 (7–342) days, respectively. During eltrombopag treatment, no side effects were encountered that required discontinuation of the drug.

In the present study, recipients of AHSCT and allo-HSCT who responded to eltrombopag had a statistically significant longer OS than those who did not respond to eltrombopag. Previously, thrombocytopenia after HSCT has been shown to be associated with TRM in the literature [2,3,20]. Currently, effective treatment options of isolated thrombocytopenia following HSCT are limited.

Thrombocyte transfusion is mandatory for the patients refractory to conventional therapies. Beside bleeding complications related to thrombocytopenia, transfusion-related complications are also important causes of mortality and morbidity [16,21]. In addition, frequent hospital admissions due to thrombocytopenia and platelet transfusions are a burden on health system [22].

Eltrombopag is promising in terms of efficacy and safety for post-HSCT thrombocytopenia treatment. Therefore, there is a need for randomized controlled studies involving large series of patients treated with eltrombopag.

## 6. Conclusion

In conclusion, eltrombopag can be used as a safe agent in appropriate patients for the treatment of thrombocytopenia after AHSCT and allo-HSCT. Although positive results have been reported in studies with a limited number and heterogeneous patient groups, such as the present study, larger randomized controlled studies are needed in order to elaborate the definitive role of eltrombopag use after HSCT.

## Figures and Tables

**Table 1:** Baseline characteristics of the patients (*)

Age, median (min-max)	53.3 (23.9–65) years
Sex, n (%)	
Male	19 (76%)
Female	6 (24%)
HSCT, n (%)	
Allo-HSCT	10 (40%)
AHSCT	15 (60%)
Primary disease requiring allo-HSCT, n (%)	
AML	3 (30%)
ALL	2 (20%)
MDS	2 (20%)
AA	1 (10%)
Non-Hodgkin lymphoma	2 (20%)
Primary disease requiring AHSCT, n (%)	
AML	2 (13.3%)
MM	8 (53.3%)
Non-Hodgkin lymphoma	3 (20%)
Hodgkin lymphoma	2 (13.3%)
Conditioning regimen (in allo-HSCT recipients)	
MAC	6 (60%)
RIC	4 (40%)
Conditioning regimen (in AHSCT recipients)	
Melphalan (for MM)	8 (53.3%)
Busulfan-cyclophosphamide (for AML)	2 (13.3)
Mitoxantrone-melphalan (for lymphoma)	3 (20)
BEAM (for lymphoma)	2 (13.3)
Donor (in allo-HSCT recipients, n = 10)	
Full matched, n (%)	7 (70 %)
Haploidentical, n (%)	3 (30%)
PIT or SFPR	
PIT	19 (76%)
SFPR	6 (24%)
Remission status, n (%)	
Remission	20 (80%)
Relapsed	5 (20%) **
CMV infection, n (%)	
Yes	2 (8%)
No	23 (92%)
GVHD (in allo-HSCT, n = 10)	
Yes	2 (20%)
No	8 (80%)

* AML: Acute myeloid leukemia, ALL: Acute lymphoblastic leukemia, MDS: Myelodysplastic syndrome, AA: Aplastic anemia, MM: Multiple myeloma, NHL: Non-Hodgkin lymphoma, HL: Hodgkin lymphoma, GVHD: Graft-versus-host disease, PIT: Prolonged isolated thrombocytopenia, SFPR: Secondary failure of platelet recovery, CMV: Cytomegalovirus, MAC: Myeloablative conditioning, RIC: Reduced intensity conditioning, BEAM: Carmustine, etoposide, cytosine arabinoside, melphalan.

** Four of these patients were lymphoma patients without bone marrow infiltration, while one of them was a multiple myeloma patient with 8% plasma cells in the bone marrow.

**Table 2:** The cumulative data of all patients receiving eltrombopag (n = 25).

Duration from HSCT to initiation of eltrombopag treatment, median (min-max)	81 (24–735) days
Follow-up time, median (min-max)	356 (20–1515) days
Eltrombopag response	
Yes	15 (60%)
No	10 (40%)
Duration from initiation of eltrombopag to response, median (min-max)	41 (7–342) days
Response dosage of eltrombopag	
75 mg	3 (30%)
100 mg	5 (33.3%)
150 mg	7 (46.75%)
Eltrombopag treatment duration of unresponsive patients, mean (±SD)	130.5 (±77) days

**Table 3:** The data of allo-HSCT and AHSCT recipients receiving eltrombopag

	Allo-HSCT (n = 10)	AHSCT (n = 15)

Remission status		
In remission	10 (100%)	10 (66.7%)
Relapsed	0 (0%)	5 (33.3%) *

SFPR	1 (10%)	5 (33.3%)

PIT	9 (90%)	10 (66.7%)

Duration from HSCT to initiation of eltrombopag treatment, mean (±SD) or median (min-max)	77.1 (±27.1) days	71 (24–735) days

Follow-up, median (min-max) or mean (±SD)	263 (84–849)	465.5 (±397.5) days

Response	5 (50%)	10 (66.7%)

Response rates in PIT and SFPR		

Response rate in SFPR, %(n)	0% (0/1)	40% (2/5)

Response rate in PIT, %(n)	55.5% (5/9)	80% (8/10)

Duration from initiation of eltrombopag to response, median (min-max)	41 (13–104) days	50 days (7–342) days

Response dosage of eltrombopag, % (n)		

75 mg	20% (1/5)	20% (2/10)

100 mg	0% (0/5)	50% (5/10)

150 mg	80% (4/5)	30% (3/10)

* Four of these patients were lymphoma patients without bone marrow infiltration, while one of them was a multiple myeloma patient with 8% plasma cells in the bone marrow

**Table 4:** Eltrombopag response rates of allo-HSCT recipients according to the parameters.

Patients	Responders, n (%)	Nonresponders, n (%)	p-value
Gender			0.44
Male	3 (60%)	5 (100%)	
Female	2 (40%)	0 (0%)	
Conditioning regimen			0.52
MAC	4 (80%)	2 (40%)	
RIC	1 (20%)	3 (60%)	
Indication of treatment			1.00
PIT	5 (100%)	4 (80%)	
SFPR	0 (0 %)	1 (20%)	
GVHD			0.44
Yes	2 (40%)	0 (0%)	
No	3 (60%)	5 (100%)	
CMV infection			1.00
Yes	1 (20%)	1 (20%)	
No	4 (80%)	4 (80%)	

* MAC: Myeloablative conditioning, RIC: Reduced intensity conditioning, PIT: Prolonged isolated thrombocytopenia, SFPR: Secondary failure of platelet recovery, GVHD: Graft-versus-host disease CMV: Cytomegalovirus.

**Table 5:** Eltrombopag response rates of AHSCT recipients according to the parameters.

Patients	Responders, n (%)	Nonresponders, n (%)	p-value
Gender			1.00
Male	7 (70%)	4 (80%)	
Female	3 (30%)	1 (20%)	
Remission status			1.00
Remission	7 (70%)	3 (60%)	
Relapsed	3 (30%)	2 (40%)	
Indication of treatment			0.25
PIT	8 (80%)	2 (40%)	
SFPR	2 (20%)	3 (60%)	

* PIT: Prolonged isolated thrombocytopenia, SFPR: Secondary failure of platelet recovery.
